# Ensuring microbial water quality for on-site water reuse: Importance of online sensors for reliable operation

**DOI:** 10.1016/j.wroa.2024.100215

**Published:** 2024-02-22

**Authors:** Eva Reynaert, Deepthi Nagappa, Jürg A. Sigrist, Eberhard Morgenroth

**Affiliations:** a Eawag, Swiss Federal Institute of Aquatic Science and Technology, 8600 Dübendorf, Switzerland; b ETH Zürich, Institute of Environmental Engineering, Zürich 8093, Switzerland; c Ashoka Trust for Research in Ecology and the Environment (ATREE), Bengaluru 560064, India

**Keywords:** On-site water reuse, Microbial water quality, Sensors, Online flow cytometry, Chlorination

## Abstract

A growing number of cities and regions are promoting or mandating on-site treatment and reuse of wastewater, which has resulted in the implementation of several thousand on-site water reuse systems on a global scale. However, there is only limited information on the (microbial) water quality from implemented systems. The focus of this study was on two best-in-class on-site water reuse systems in Bengaluru, India, which typically met the local water quality requirements during monthly compliance testing. This study aimed to (i) assess the microbial quality of the reclaimed water at a high temporal resolution (daily or every 15 min), and (ii) explore whether measurements from commercially available sensors can be used to improve the operation of such systems. The monitoring campaign revealed high variations in microbial water quality, even in these best-in-class systems, rendering the water inadequate for the intended reuse applications (toilet flushing and landscape irrigation). These variations were attributed to two key factors: (1) the low frequency of chlorination, and (2) fluctuations of the chlorine demand of the water, in particular of ammonium concentrations. Such fluctuations are likely inherent to on-site systems, which rely on a low level of process control. The monitoring campaign showed that the microbial water quality was most closely related to oxidation–reduction potential (ORP) and free chlorine sensors. Due to its relatively low cost and low need for maintenance, the ORP emerges as a compelling candidate for automating the chlorination to effectively manage variations in chlorine demand and ensure safe water reuse. Overall, this study underscores the necessity of integrating treatment trains, operation, and monitoring for safe on-site water reuse.

## Highlights

• Study focus: Monitor two best-in-class non-potable water reuse systems.
• Monitoring revealed high variability of microbial water quality from day to day.
• This variability mostly goes unseen with monthly compliance verification.
• Variability stems from ineffective chlorination due to fluctuating chlorine demand.
• ORP sensors could be used to automate chlorination to adapt to fluctuations.

## Graphical abstract

**Figure:**
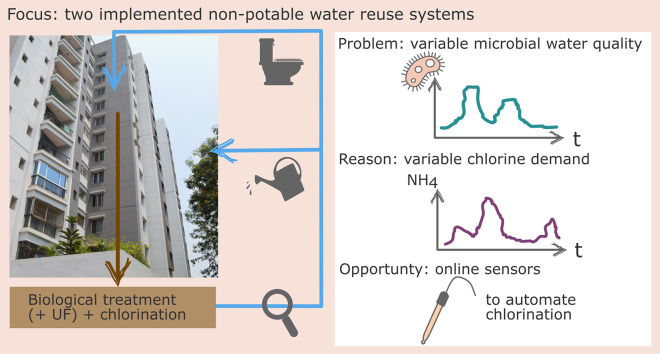

## 1 Introduction

Water scarcity constitutes a major threat to human well-being, economic development and ecosystem health (Gleick and Cooley, 2021). To mitigate the impacts of water scarcity and reduce environmental pollution caused by untreated wastewater, an increasing number of cities and regions are promoting or even mandating on-site treatment and reuse of wastewater (Goyal and Kumar, 2022; Lackey et al., 2020; Neighbour and Qi, 2018). These systems are typically implemented at the scale of a building, and reclaimed water is used for non-potable applications such as toilet flushing or landscape irrigation. Cities with large numbers of implemented on-site non-potable water systems (ONWS) include Beijing, China, with two to three thousand ONWS (Binz and Truffer, 2017) and Bengaluru, India, with more than three thousand ONWS (Klinger et al., 2020). In Beijing, the construction of ONWS is driven by a regulation requiring that all institutes, hotels, schools and residences with areas exceeding 30′000 m^2^ install such systems (Liang and van Dijk, 2010), while in Bengaluru, a local mandate requires the treatment and reuse of all wastewater on the premises for residential developments with 20 or more units or a built-up area of 2′000 m^2^ or more (BWSSB, 2018).

Various technologies exists that are able to produce high-quality reclaimed water (Salgot and Folch, 2018). A scoping study from India assessing almost 300 sanitation systems serving 10–1′000 households showed that the majority of implemented ONWS used traditional technologies such as conventional activated sludge (CAS) or sequencing batch reactors (SBRs) for main treatment. Chlorination was implemented in all systems that had final disinfection (Klinger et al., 2020).

Ensuring that the microbial quality of the reclaimed water from ONWS is acceptable at all times is critical, as users are routinely exposed to small quantities of the water, e.g., through ingestion after dermal contact or through the inhalation of aerosols (Schoen et al., 2017, 2020). One challenge for ONWS is that proper operation and maintenance need to be ensured to consistently produce microbially safe reclaimed water. Recent studies have demonstrated that even brief treatment failures can substantially decrease the average treatment performance of water reuse systems, thereby increasing the risk to human health (Schoen et al., 2018; Soller et al., 2018). Understanding temporal variations in microbial water quality in on-site systems is critical to assess health risks from using reclaimed water. There are some reports indicating that many implemented ONWS are not well operated (Klinger et al., 2020; Kuttuva et al., 2018; Neighbour and Qi, 2018). However, there is a scarcity of rigorous academic studies on water quality from implemented ONWS, resulting in a discrepancy between the available body of literature and practical implementation (Wilcox et al., 2016).

Current studies on ONWS predominantly focus on field tests of novel technologies such as membrane-based systems or technologies using electrochemical treatment (e.g., Sutherland et al., 2021; Varigala et al., 2020; Welling et al., 2020). While technology field tests offer valuable insights on the feasibility of such treatment, they typically involve highly skilled operators that ensure excellent maintenance and are therefore not representative of real-world operation. Conversely, most literature examining water quality from systems using traditional technologies, such as CAS, centers on large-scale centralized systems and on discharge. However, on-site systems face a range of operational challenges that are specific to their small scale. These challenges include (i) the reliance on relatively untrained personnel for operation (Klinger et al., 2020; Neighbour and Qi, 2018), (ii) high cost for maintenance due to the lack of economies of scale (Massoud et al., 2009), (iii) potentially greater variations of the influent wastewater composition (Schneider et al., 2020), and (iv) the lack of monitoring (Eggimann et al., 2017).

Despite the existence of several thousand ONWS globally, this discrepancy between research and implementation has resulted in a gap, where there is limited information on the temporal variability of the (microbial) water quality in implemented systems. Data is particularly scarce when looking beyond the concentration of fecal indicator bacteria in the reclaimed water, encompassing the removal of other groups of pathogen indicators, in particular viruses, or the regrowth of pathogens in the reclaimed water.

In this study, we closely monitored the microbial quality (removal of bacteria and viruses, and regrowth of bacteria) in the reclaimed water from two implemented on-site systems in Bengaluru, India, at a high temporal resolution (daily or every 15 min). We further explored whether operation of on-site systems can be improved using measurements from commercially available sensors.

## 2 Results and discussion

### 2.1 Focus of the study: best-in-class implemented on-site water reuse systems

Ranked among the fastest growing cities in the world, Bengaluru, India, has faced difficulties in creating new water and wastewater infrastructure to meet the growing needs of its population (Raj, 2016). To reduce freshwater consumption and avoid environmental pollution from untreated wastewater, the local environmental protection agency KSPCB has mandated large apartment complexes to treat and reuse all wastewater within the complex premises, leading to the implementation of at least 3′000 ONWS across the city (Klinger et al., 2020). However, there is evidence that the large majority of ONWS fails to meet the water quality requirements (Kuttuva et al., 2018). This study focuses on two implemented systems, ONWS#1 and ONWS#2, that typically comply with the legal requirements during monthly verification testing (detailed requirement in Section 4.1) and are thus considered as best-in-class ONWS. The rationale for focusing on best-in-class ONWS lied in our interest to explore (i) the suitability of monthly water quality sampling for providing reliable information on performance over a month, and (ii) the variability in treatment performance of well-designed treatment trains, where simple operational measures may be implemented to improve water quality, as opposed to non-performing systems that may require significant redesign. The ONWS were representative of well-designed and well-operated treatment trains in Bengaluru. ONWS#1 used CAS, followed by pressure sand and granular activated carbon filters, and finally chlorination to produce water for toilet flushing. ONWS#2 produced water of two qualities. One type of reclaimed water was reused for landscaping (ONWS#2-LS) after treatment with SBR, pressure sand and granular activated carbon filters, and chlorination. The second type was reclaimed water for toilet flushing (ONWS#2-TF), which underwent further treatment with ultrafiltration and chlorination. In ONWS#1 and ONWS#2-TF, concentrated hypochlorite solution was added through dosing pumps that fed into the pipes through which water was transported into the chlorination tanks once to twice per day. In ONWS#2-LS, chlorine was manually added directly into the chlorination tank daily (see Section 4.1). For the testing, water from the chlorination tanks was recirculated through flow cells with five commercially available online sensors hypothesized to be linked with microbial water quality (Section 4.2.1). Manual samples for offline analysis of the physico-chemical (Section 4.2.2) and microbial (Section 4.3.2) water quality were collected from the same locations.

### 2.2 Short-term variability of the microbial water quality

**Fig. 1:**
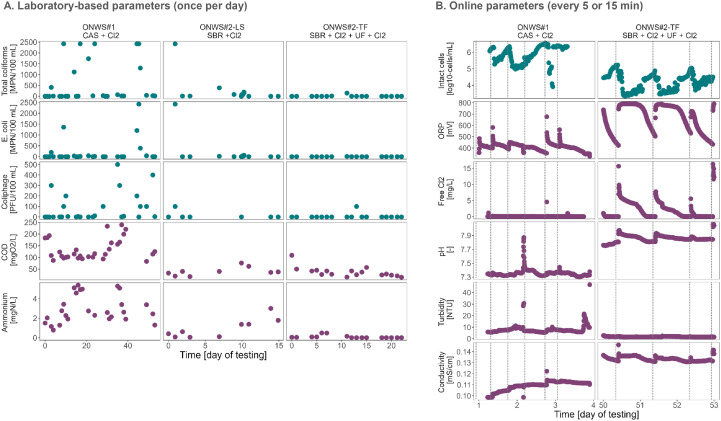
Microbial (turquoise), and physicochemical (purple) quality of reclaimed water from on-site non-potable water systems (ONWS) to be reused for toilet flushing (ONWS#1, ONWS#2-TF) or landscape irrigation (ONWS#2-LS). The time is relative to the first day of sampling in the specified system. CAS: conventional activated sludge. Cl_2_: chlorine. SBR: sequencing batch reactor. UF: ultrafiltration. MPN: most probable number. PFU: plaque forming units. The upper limit of quantification for total coliforms and E. coli was 2′400 MPN / 100 mL. Plot B is depicted only over 3 day periods to highlight daily patterns (no data for ONWS#2-LS). Dotted vertical lines: chlorine additions (every ∼12 h in ONWS#1, and every ∼24 h in ONWS#2-TF, over the full testing period).

Measurements of three microbial water quality indicators, namely *E. coli*, total coliforms, and somatic coliphages, revealed that there were significant variations in microbial water quality on a daily basis in all reclaimed waters (Fig. 1A).

Generally, ONWS#2-TF (with ultrafiltration membrane) had a markedly improved water quality compared to ONWS#1 and ONWS#2-LS, with microbial indicator concentrations mostly falling below the limits of detection. For all three treatment trains, however, there were events with high concentrations of total coliforms and *E. coli*, with the most probable numbers (MPN) sometimes exceeding 2′400 / 100 mL, and coliphage concentrations of up to 500 plaque forming units (PFU) / 100 mL.

Ensuring user health requires not only the removal of pathogenic microorganisms from the wastewater (assessed through measurements of *E. coli*, total coliforms and coliphages), but also the prevention of bacterial (re)growth in the reclaimed water. Through the use of online flow cytometry, we were able to monitor the intact cell concentration (ICC) every 15 min in the two tanks containing reclaimed water to be reused for toilet flushing (ONWS#1 and ONWS#2-TF) for periods of 6 days in ONWS#1 (with interruptions, total of 248 data points), and 7 days in ONWS#2-TF (without interruptions, 698 data points). The ICC fluctuated over several orders of magnitudes: in ONWS#1, the log_10_-concentration of intact cells (per mL) varied between 4 and 6.5, while concentrations ranged between 3 and 5.5 in ONWS#2-TF (Fig. 1B). The ICC does not directly inform on the presence/absence of pathogens in the reclaimed water. However, it has demonstrated its utility as an indicator of process performance (Cheswick et al., 2019; Van Nevel et al., 2017), and has in particular been shown to descriptively quantify changes in cell concentrations in chlorination processes (Ziemba et al., 2019).

#### 2.2.1 Monthly verification testing is not sufficient to ensure microbial water quality

The observed variations in microbial water quality are problematic for safe reuse of the reclaimed water. A comparison with requirements from 18 water reuse frameworks (guidelines, regulations, etc.) shows that none of the ONWS consistently met the maximum concentrations in reclaimed water for toilet flushing for *E. coli* and total coliforms (overview of frameworks in Reynaert et al., 2020; relevant requirements presented in Supplementary Information 1). The monitoring campaign therefore demonstrates that monthly sampling of microbial indicators is inadequate to ensure that microbial water quality requirements are met at all times, as a large part of the variability goes unseen at such a low sampling frequency.

### 2.3 Reasons for the variability in microbial water quality

#### 2.3.1 Fluctuations of physico-chemical water quality result in variable chlorine demand

The monitoring campaign showed that there were also major variations in the chemical water quality (Fig. 1A). In ONWS#1, the chemical oxygen demand (COD) in the reclaimed water varied between 85 and 240 mg_O2_/L, while the ammonium concentration ranged between 0.8 and 5.4 mg_N_/L. The reclaimed water was generally of higher quality in ONWS#2, but COD and ammonium concentrations were also variable: In ONWS#2-LS, the COD varied between 20 and 75 mg_O2_/L and ammonium between 0.05 and 3 mg_N_/L, while the COD ranged between 15 and 110 mg_O2_/L and the ammonium between 0.01 to 0.5 mg_N_/L in ONWS#2-TF.

Short-term variations of organics and ammonium concentrations are a challenge for the control of chlorination. Chlorine disinfection is used to inactivate pathogens from the wastewater and to provide ongoing protection against regrowth of pathogens by maintaining a chlorine residual in the reclaimed water. In the presence of ammonium, free chlorine (uncombined chlorine in the form of HOCl or OCl^−^) will rapidly form chloramines, which also act as disinfectants, but are much less effective than free chlorine (Ikehata et al., 2018). At high ratios of chlorine to ammonium, chlorine is consumed at a ratio of 7.6 mg of chlorine per mg of ammonium (Pressley et al., 1972), implying that even small variations of ammonium concentrations result in large variations of the chlorine demand. Free chlorine can also react with organic compounds, which – besides making the chlorine unavailable for disinfection – can result in the formation of toxic disinfection by-products (Sedlak and von Gunten, 2011). The added chlorine dose must therefore meet the chlorine demand of the water, in order to produce a chlorine residual, while minimizing the production of by-products (Clark and Sivaganesan, 2002; Sedlak and von Gunten, 2011).

#### 2.3.2 The variability of chlorine demand in on-site system can likely be reduced, but not eliminated

The operation of the two ONWS could likely be improved to achieve lower ammonium and COD concentrations in the reclaimed water, in particular by addressing insufficient aeration in ONWS#1. However, part of the variability in chemical water quality is likely inherent to the operation of on-site treatment systems. Several studies investigating the water quality from implemented on-site wastewater treatment systems (including membrane bioreactors, CAS, and SBRs) have documented significant temporal variability in the ammonium and frequently also in the organic effluent concentrations (Abegglen et al., 2008; Hanna et al., 1995; Klinger et al., 2020; Schneider et al., 2020).

One reason for the high variability of the physicochemical water quality in these implemented systems is likely the low level of control and automation, rendering on-site systems unable to flexibly react to variations in the influent composition and other changes in conditions. In particular, none of the studied systems controlled the aeration rate, which is a critical parameter for stable nitrification. Dissolved oxygen sensors, as typically implemented for aeration control in large-scale plants (Newhart et al., 2020), incur significant costs and require regular maintenance, challenging their integration into on-site systems. Providing that the aeration rate is reasonably well-tuned, it may be simpler to automate the chlorination to mitigate the effects of variations in chlorine demand.

### 2.4 Correlations between microbial water quality and sensor measurements

Five commercially available online sensors were installed in the chlorination tanks containing the reclaimed water to be reused for toilet flushing (ONWS#1 and ONWS#2-TF) to assess the sensors’ capacity to provide information on the microbial quality of the water. ORP, free chlorine, and pH sensor measurements closely resembled the pattern of chlorine additions (Fig. 1B). In ONWS#2-TF, the ORP increased to up to 800 mV after chlorine addition, while free chlorine concentrations could be as high as 15 mg/L and decreased to around 400 mV and 0 mg/L before new chlorine was added, respectively. The pH and conductivity also responded briefly to chlorination events, as chlorine was added in the form of a sodium hypochlorite solution (pH 12). In contrast, the ORP was rarely above 600 mV in ONWS#1, and free chlorine was mostly absent, due to the high concentrations of ammonium and organics in the water.

#### 2.4.1 ORP and free chlorine are most closely correlated with several microbial water quality indicators

**Fig. 2:**
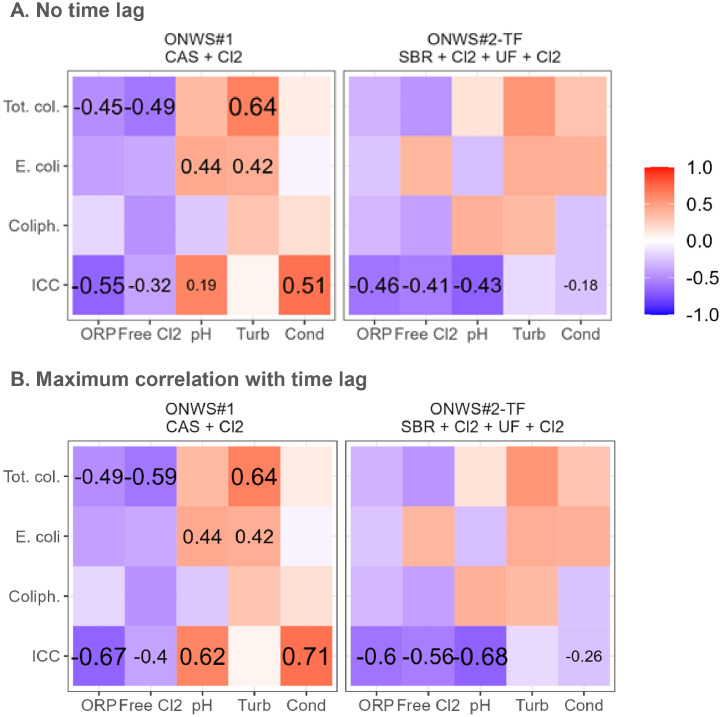
Spearman rank correlation computed without time lag (A) and the maximum lagged correlation (correlations computed for time lags ranging from 0 min to 6 h in 5 min steps) (B). Numbers are only shown for correlations with p-value < 0.05. ORP: oxidation–reduction potential; Turb: turbidity; Cond: conductivity.

A good predictor for microbial water quality should (i) be correlated with several microbial water quality indicators, and (ii) have consistent correlations between different ONWS (Reynaert et al., 2023a). A correlation analysis shows that the ORP and free chlorine sensors are the most promising candidates, as they are correlated with both, fecal indicator organisms and ICC, and show consistent correlations in ONWS#1 and ONWS#2-TF (Fig. 2A). Note that none of the sensors was significantly correlated with any of the fecal indicator organisms in ONWS#2-TF, as the dataset had a high fraction of non-detects (coliphage) and fecal indicator bacteria were consistently removed by the ultrafiltration membrane (*E. coli* and total coliforms). In contrast to ORP and free chlorine, pH and conductivity had opposite correlations in ONWS#1 and ONWS#2-TF, and turbidity was not correlated with the ICC in either system. These correlations are also visually supported by Fig. 1B, where ORP and free chlorine are related to chlorination events and to the ICC, while turbidity, pH and conductivity do not follow consistent patterns.

Comparing ORP and free chlorine, we see that correlations are similar for total coliforms and *E. coli* (ONWS#1). However, there is a major difference for ICC, especially in ONWS#1, which was characterized by higher levels of ammonium. This stronger correlation for ORP can be explained by considering that ORP (i) reflects the disinfection capacity of all chlorine species, including chloramines, rather than solely the most reactive species represented by free chlorine, and (ii) accounts for the speciation of the free chlorine as a function of temperature and pH (Victorin et al., 1972). The equivalent or superior performance of ORP compared to free chlorine to operate and monitor ONWS has also already been reported for laboratory-based studies studying membrane bioreactors with chlorination (Reynaert et al., 2023a, 2023b). The present work extends these previous studies by confirming the results for implemented ONWS encompassing different treatment trains.

#### 2.4.2 Considering the time lag between sensor measurements and microbial water quality allows for a better comparison of sensors

The correlations from Fig. 2A are based on sensor and microbial water quality measurements collected at the same moment in time. However, there is likely a time lag between changes in physicochemical water quality and resulting changes in microbial water quality. For instance, it takes time for bacteria to regrow in the reclaimed water after chlorine concentrations have decreased (see Fig. 1B). A cross-correlation analysis shows that time-lagged correlations are indeed higher for the ICC (Fig. 2B). ORP and free chlorine have the highest correlation with the ICC at a time lag of 60 min (ONWS#1), respectively 150 min (ONWS#2-TF), and with total coliforms and *E. coli* after around 30 min (ONWS#1) (see Supplementary Information 2). Note that there should not be such time lags under normal operation in well-operated systems, as the aim is to ensure stable microbial water quality. In this study, however, the time lag analysis allows us to reduce the effect of the dynamics of chlorination to allow for a better comparison of sensors.

### 2.5 Way forward

The variability of the chlorine demand is likely inherent in on-site systems, as a result of the way in which they are operated. As a consequence, the chlorine dosing will need to be able to flexibly adapt to such variations through the automation of the chlorination. The correlation analysis showed that the ORP is an interesting candidate to automate chlorination in on-site systems, as it provides information on the efficacy of chlorination in removing microbial indicators, while being relatively inexpensive and requiring little maintenance. In comparison, commercially available chlorine sensors are substantially more costly and require more frequent maintenance (Herold et al., 2023).

#### 2.5.1 Automating chlorination with sensors setpoints

One important question revolves around determining appropriate ORP setpoints to automate the chlorination. Unfortunately, most water reuse frameworks do not provide guidance on ORP setpoints for water reuse (Reynaert et al., 2021). Reynaert et al. (2023a) proposed an approach to set setpoints ensuring different microbial water qualities in membrane bioreactors with chlorination by operating systems with constant (chlorination) settings for extended periods of time before sampling the microbial water quality. This operational approach allowed to mitigate system dynamics. The data collection in the present study, which aimed at observing dynamics of microbial water quality under actual operation, is not optimized for the determination of sensor setpoints. Preliminary results taking the time lag into account (i.e., comparing the microbial water quality with sensor data for which the correlation is highest) indicate that ORP setpoints calculated using the present dataset are in the same order as those presented in (Reynaert et al., 2023a) (see Supplementary Information 3). However, the validation of these setpoints will require an adequate operation of ONWS with stable quality of the reclaimed water.

#### 2.5.2 ORP-based chlorination control has proved its value in other applications

ORP control of chlorination is not a novel concept and has been tested and implemented in full-scale wastewater treatment plants (WERF, 2004). However, these studies lack comprehensive results regarding microbial water quality, in particular in terms of virus removal and bacterial regrowth, due to their focus on discharge rather than reuse. Another example is chlorination control using ORP implemented in swimming pool disinfection, for which some studies have shown that ORP is a better predictor of water quality than free chlorine (Bastian and Brondum, 2009; Steininger, 1991). Swimming pool chlorination is more similar in scale to the systems described in this study but treats a substantially different water matrix that will normally not contain high concentrations of enteric pathogens. A practical challenge for ORP-based chlorination control may be the relatively slow reactivity of ORP sensors. This could be a problem for ONWS that batch-chlorinate, requiring the addition of large quantities of chlorine in a short time frame. There is therefore value in further development and testing of ORP-based chlorination control in (implemented) on-site water reuse systems. Such testing will provide the basis to verify that automated chlorine dosage results in stable chlorine residual, and, ultimately, improved microbial water quality.

#### 2.5.3 Policy implications

In light of the widespread use of chlorination for disinfection globally and in Bengaluru, it will be critical to provide guidance and requirements for the implementation of chlorination in ONWS to ensure the protection of user health. Overall, this study underscores the necessity of addressing technologies, operation, and monitoring of ONWS jointly. In particular, the results highlight the inadequacy of periodic monitoring through grab samples in providing reliable information on water quality between sampling events. In the long term, the efficiency and effectiveness of microbial water quality monitoring can be improved through the use of sensors for remote monitoring, allowing for automated alarms if treatment targets are not met. However, the implementation of remote monitoring requires addressing the existing system challenges first, specifically by (i) operating the biological treatment to prioritize low and stable ammonium concentrations, and (ii) automating the chlorination to manage the residual variability in chlorine demand.

## 3 Conclusions

• The present study revealed several instances with inadequate microbial water quality in two implemented ONWS in Bengaluru, India, as a result of ineffective chlorination. Both ONWS were best-in-class systems that usually complied with legal water quality requirements during monthly testing, demonstrating that such low testing frequencies are insufficient to ensure that requirements are met at all times.
• Fluctuations of the physicochemical water quality, especially in terms of ammonium, in reclaimed water from implemented ONWS challenges the efficacy of chlorine disinfection. Given the inherent variability of chlorine demand in on-site systems, automating chlorine dosage based on sensor feedback is necessary to ensure reliable disinfection.
• Monitoring results demonstrate a strong correlation between ORP and the microbial water quality, especially when considering a lag period between sensor measurements and changes in microbial water quality.
• There is need for regulators to define ORP setpoints for on-site systems, where ensuring the microbial water quality at all times is critical.

## 4 Materials and methods

### 4.1 On-site water reuse systems

The reclaimed water quality of two ONWS located within apartment complexes in Bengaluru, India, was studied for two months each, between September and December 2022. The focus of this study was set on well-performing ONWS, which would typically meet the water quality requirements in the prescribed monthly samples. The local environment protection agency KSPCB requires monthly analyses of seven parameters, namely the pH (6.5–9), biochemical oxygen demand after 5 days (BOD_5_ ≤ 10 mg/L), COD (≤ 50 mg/L), total suspended solids (TSS ≤ 20 mg/L), ammonia nitrogen (≤ 5 mg/L), total nitrogen (≤ 10 mg/L) and fecal coliforms (≤ 100 MPN/100 mL), in the reclaimed water by nationally-accredited laboratories.

Both apartment complexes had a dual piping system to reuse water for toilet flushing, and were surrounded by communal green spaces, where reclaimed water was used for irrigation. Details on the ONWS are presented in Table 1. Teams of two or three operators were responsible for the operation, to manually turn on / off pumps, and take daily measurements of the sludge volume and chlorine concentration. In both systems, the reclaimed water was initially stored in a storage tank for landscaping water (not chlorinated in ONWS#1, chlorinated through the addition of variable amounts of sodium hypochlorite in ONWS#2), from where it was pumped directly into the chlorination tank holding the toilet flush water (ONWS#1), or through an ultrafiltration membraned and then into the chlorination tank (ONWS#2) once to twice per day. In both plants, the chlorine was added through a dosing pump that fed into the pipe through which water was transported into the chlorination tank. The chlorine dosing pump was manually turned on whenever a fresh batch of water entered the toilet flush water storage tank.

**Table 1:** Treatment capacity, treatment steps and reuse applications for the two onsite non-potable water systems (ONWS).

	ONWS#1	ONWS#2
Year of commissioning	2010	2011
Treatment capacity	550 m^3^/d	200 m^3^/d
Connected apartments/residents	509 apartments, 2′545 residents	156 apartments, 780 residents
Treatment and reuse applications	1. Bar screen; 2. Oil & grease trap; 3. Conventional activated sludge process; 4. Pressure sand filter; 5. Granular activated carbon filter → *Reuse for landscaping;* 6. Disinfection with sodium hypochlorite → *Reuse for toilet flushing*	1. Bar screen; 2. Oil & grease trap; 3. Conventional activated sludge process; 4. Pressure sand filter; 5. Granular activated carbon filter; 6. Disinfection with sodium hypochlorite → *Reuse for landscaping* 7. Ultrafiltration; 8. Disinfection with sodium hypochlorite → *Reuse for toilet flushing*

### 4.2 Physicochemical water quality

#### 4.2.1 Online measurements

Water from the toilet flush water storage tank was constantly recirculated through flow cells equipped with five online sensors with an assumed mechanistic relationship with microbial water quality: ORP, free chlorine, pH, turbidity and conductivity (details in Table 2). Reference measurements were taken with the recommended buffer solutions for ORP (220 mV) and pH (pH 4 and 7). The FC sensor was calibrated with reference free chlorine measurements (Hach DPD test kits, 0–2 mg/L free chlorine, Hach, Loveland, USA) at the flow cell. Sensors measurements were automatically logged at 5-min intervals.

**Table 2:** Specifications and expected link to microbial water quality of the sensors installed in the chlorination tanks containing the reclaimed toilet flush water. All sensors were purchased from Endress+Hauser, Reinach, Switzerland.

Measurement	Sensor specification	Mechanistic relationship with the microbial water quality
Oxidation-reduction potential (ORP)	Ceragel CPS72D	Measurement of the oxidizing or reducing tendency of the water. For chlorinated systems: oxidative capacity of all chlorine species James et al. (2004)
Free chlorine (FC)	Digital free chlorine sensor Memosens CCS51D	Free chlorine is the most effective chlorine form when disinfection with sodium hypochlorite Kim and Hensley (1997)
pH	Orbisint CPS11D	Information on speciation and thus, disinfection potential of free chlorine Cherney et al. (2006)
Turbidity	Turbimax CUS52D	Turbidity can be linked to bacteria concentrations Hess et al. (2021)
Conductivity	Condumax CLS21D	The addition of sodium hypochlorite solution is linked to an increase in conductivity

#### 4.2.2 Offline measurements

Grab samples of the wastewater influent and of the reclaimed water (landscaping water or toilet flush water storage tanks) were collected daily (on weekdays) for a period of two months in each ONWS. Duplicate samples were measured for TSS, BOD_5_ and COD using APHA Standard Methods 2540D, 5210B and 5220B, respectively. Samples for TSS and BOD_5_ were analyzed on the same day as sample collection. Samples for COD analysis were preserved at a pH <2 using sulfuric acid and stored at 4 °C before analysis on a bi-weekly basis.

Concentrations of total nitrogen, nitrate and ammonium were determined photometrically using a Spectroquant® Prove 600 (Merck KGaA, Darmstadt, Germany) spectrophotometer and corresponding test kits. All samples were filtered using a 0.45 µm pre-rinsed filter paper (Durapore® Membrane Filter, 0.45 µm, HVLP04700, Merck Millipore) and analyzed on the same day as sample collection. Influent samples for total nitrogen and ammonium were frequently diluted 1:5 and seldom 1:10, and those of the treated effluent were mostly undiluted and seldom diluted 1:2. For total nitrogen, the samples were then digested using a Spectroquant® Crack Set 20 and analyzed for concentration of nitrate-nitrogen in the range of 0.1–25.0 mg/L (Kit No. 109,713). For ammonium, samples were analyzed using a test kit in the range of 0.010–3.00 mg/L (Kit No. 114,652).

Free and total chlorine were measured immediately after sampling using a portable spectrophotometer (DR 1900, Hach, Loveland, USA) with corresponding test kits (DPD, 0–2 mg/L free chlorine, Hach, Loveland, USA).

The full dataset with concentrations of all physicochemical water quality parameters in the wastewater and in the reclaimed water is available at https://doi.org/10.25678/000CCT.

### 4.3 Microbial water quality

#### 4.3.1 Online measurements

The ICC measured by online flow cytometry was used to indicate the ability of bacteria to survive and grow during storage. We used a custom-made automated sampling, staining and incubation module combined with an Accuri C6 flow cytometer (BD Accuri, San Jose, CA, United States) to draw and analyze water samples from the reclaimed water storage tanks (Besmer and Hammes, 2016). Water samples were stained with SYBR® Green I fluorescent stain (ThermoFisher Scientific, Waltham, Massachusetts, USA, final concentration 1:10′000) and propidium iodide (ThermoFisher Scientific, Waltham, Massachusetts, USA, final concentration 6 µM) and incubated for 12 min at 37 °C (Nescerecka et al., 2016). 100 µL of sample were analyzed every 15 min. After each cycle, the staining module was rinsed with deionized water. An additional automated cleaning cycle with sodium hypochlorite and soap was conducted once per day.

#### 4.3.2 Offline measurement

A set of grab samples from the wastewater influent and treated effluent were collected daily (on weekdays) in sterile borosilicate glass bottles (100 mL) at different times of the day. Total coliforms and *E. coli* were used as indicators for the removal of enteric bacteria from the wastewater. Collected samples were cooled for transport and analyzed immediately in the laboratory using an enzyme activity test (Colilert 18/Quanti-Tray, IDEXX Laboratories, Westbrook, USA).

We used somatic coliphages as indicators for the removal of enteric viruses. The double agar layer was used to enumerate the infectious bacteriophage as plaque forming units (PFU) following the ISO 10,705–2 method, with *E. coli* strain C (ATCC 13,706).

The full dataset with concentrations of microbial water quality indicators in the wastewater and in the reclaimed water is available at https://doi.org/10.25678/000CCT.

### 4.4 Correlation analyses

Spearman's rank correlation was employed to quantify the association between sensor measurements and microbial water quality, accounting for possible non-linear relationships. Observations were paired based on their timestamps. In the time lag analysis, the timestamps of the sensor measurements and microbial water quality were shifted by the time lag; for instance with a time lag of 60 min, the microbial water quality data was paired with sensor measurements taken 60 min earlier.

## CRediT authorship contribution statement

**Eva Reynaert:** Conceptualization, Formal analysis, Investigation, Methodology, Visualization, Writing – original draft, Writing – review & editing. **Deepthi Nagappa:** Investigation, Methodology, Writing – review & editing. **Jürg A. Sigrist:** Methodology, Writing – review & editing. **Eberhard Morgenroth:** Conceptualization, Writing – original draft, Writing – review & editing.

## Declaration of competing interest

The authors declare that they have no known competing financial interests or personal relationships that could have appeared to influence the work reported in this paper.

## Data Availability

The full dataset with concentrations of microbial water quality indicators in the wastewater and in the reclaimed water are available from https://doi.org/10.25678/000CCT.

## Appendix Supplementary materials

Image, application 1
