# Supplementary material for: Ensuring microbial water quality for on-site water reuse: Importance of online sensors for reliable operation
Source: Water Res X. 2024 Feb 22;22:100215. doi: 10.1016/j.wroa.2024.100215 (PMC11144787; doi:10.1016/j.wroa.2024.100215)
Supplement: Supplementary file 1 [file mmc1.docx]

Ensuring Microbial Water Quality for On-site Water Reuse: Importance of Online Sensors for Reliable Operation

Eva Reynaert^a,b^, Deepthi Nagappa^c^, Jürg A. Sigrist^a^, Eberhard Morgenroth^a,b,*^

^a^ Eawag, Swiss Federal Institute of Aquatic Science and Technology, 8600 Dübendorf, Switzerland

^b^ ETH Zürich, Institute of Environmental Engineering, 8093 Zürich, Switzerland

^c^ Ashoka Trust for Research in Ecology and the Environment (ATREE), 560064 Bengaluru, India

Corresponding author: [eberhard.morgenroth@eawag.ch](mailto:eberhard.morgenroth@eawag.ch)

**Supplementary Information 1**: Overview of International Water Reuse Frameworks Applicable to Reuse for Toilet Flushing

**Supplementary Information 2**: Time Lag Analysis

**Supplementary Information 3**: Logistic Regression Analysis

**Supplementary Information 1: Overview of International Water Reuse Frameworks Applicable to Reuse for Toilet Flushing**

**Table 1**. Overview of several frameworks for wastewater reuse for toilet flushing as presented by Reynaert et al. (2020). ND: not determined. CFU: colony-forming unit. MPN: most probable number. PFU: plate-forming unit. min, max, med, geom: minimum, maximum, median, geometric mean. wk, mon, ann: weekly, monthly, annual.

| **Water Reuse Framework** | **Country/**  **State** | **Reuse purpose** | **Residual chlorine (mgCl_2_/L)** | **E.coli (MPN or CFU/100mL)** | **Faecal coliforms (MPN or CFU/100mL)** | **Total coliforms (MPN or CFU/100mL)** | **Coliphage MS2 (PFU/100mL, LRV)** |
| --- | --- | --- | --- | --- | --- | --- | --- |
| International Standardization Organization (2018): ISO 30500: Non-Sewered Sanitation Systems -- Prefabricated Integrated Treatment Units -- General Safety and Performance Requirements for Design and Testing. | International | Toilet flushing | n/a | <= 1 CFU >= 6 LRV | n/a | n/a | <= 1 >=7 LRV |
| California State Water Resources Control Board (2018): Regulations Related to Recycled Water (Title 22). | USA, California | Toilet flushing | n/a | n/a | n/a | <= 2.2 MPN (7 med) <= 23 MPN (one sample of 30d) <= 240 (max) | >= 5 LRV (or poliovirus) |
| United States Environmental Protection Agency (2012): Guidelines for Water Reuse. | USA,  Arizona | Unrestricted urban reuse | n/a | n/a | ND in least 4 of 7 samples  <= 23 MPN (max) | n/a | n/a |
|  | USA, California | Unrestricted urban reuse | n/a | n/a | n/a | <= 2.2 MPN (7-day med) <= 23 MPN (not more than one sample exceeds this value in 30 d) <= 240 MPN (max) | n/a |
|  | USA, Florida | Unrestricted urban reuse | > 1 |  | 75 % of samples ND <= 25 MPN (max) | n/a | n/a |
|  | USA, Hawaii | Unrestricted urban reuse | > 5 |  | <= 2.2 MPN (7d median) <= 23 MPN (not more than 1 sample exceeds this in 30d) <= 200 MPN (max) | n/a | n/a |
|  | USA, Nevada | Unrestricted urban reuse | n/a | n/a | n/a | <= 2.2 MPN (30d geom) <= 23 MPN (max) | n/a |
|  | USA, New Jersey | Unrestricted urban reuse | n/a | n/a | <= 2.2 MPN (wk med) <=14 MPN (max) | n/a | n/a |
|  | USA, North Carolina | Unrestricted urban reuse | n/a | <=14 MPN (mon mean)  <= 25 MPN (max) | <= 14 MPN (mon mean) <= 25 MPN (max) | n/a | n/a |
|  | USA, Texas | Unrestricted urban reuse | n/a | <= 20 MPN (30d geom)  <= 75 MPN (max) | <= 20 MPN (30d geom)  <= 75 MPN (max) | n/a | n/a |
|  | USA, Virginia | Unrestricted urban reuse | > 1 | <= 11 MPN (mon geom) <= 35 MPN (max) | <= 14 MPN (mon geom) <= 49 MPN (max) | n/a | n/a |
|  | USA, Washington | Unrestricted urban reuse | > 1 | n/a | n/a | <= 23 MPN (7d-med) <=240 (max) | n/a |
|  | Recommen-dation of US EPA | Unrestricted urban reuse | n/a | n/a | n/a | n/a | n/a |
| Environmental Health Directorate of Western Australia (2010): Code of Practice for the Reuse of Greywater. Guidelines for the Non-potable uses of recycled water. | Australia, Western Australia | Toilet flushing | 0.2-2.0 | < 1 (MPN or CFU, med of 6 consecutive samples) | n/a | n/a | <1 (med of 6 consecutive samples) |
| Ministry of Health Canada (2010): Canadian Guidelines for Domestic Reclaimed Water for Use in Toilet and Urinal flushing. | Canada | Toilet flushing | >= 0.5 | ND (med) <= 200 CFU (max) | n/a | n/a | n/a |
| Ministry of the Environment and Rural and Marine Affaires Spain (2007): RD 1620/2007: Spanish Regulations for Water Reuse. | Spain | Sanitary appliances | n/a | ND | n/a | n/a | n/a |
| Ministry of the Environment and the Protection of the Territory Italy (2003): DM 185/2003: Regulation Containing Technical Rules for the Reuse of Wastewater. | Italy | Toilet flushing | n/a | <= 10 CFU (80% of samples) <= 100 CFU (max) | n/a | n/a | n/a |
| State Administration of Quality Supervision, Inspection and Quarantine China (2002): Municipal Wastewater Recycling: Water Quality Standards for Urban Water Consumption (misc.). | China | Toilet flushing | >= 1 (after 30 min) >= 0.2 (in pipe) | n/a | n/a | <= 0.3 (<3 units/L) | n/a |
| Environmental field testing platform South Africa (2019): Standard for Water to be Recycled as Flushwater from Prototypes on the EFT. | South Africa | Toilet flushing and hand washing | > 0.5 (avg) | 1 (avg) 10 (max) | n/a | n/a | n/a |

**Supplementary Information 2: Time Lag Analysis**

Figure 1 presents a time lag analysis of the Spearman rank correlation for time lags between 0 and 360 min.


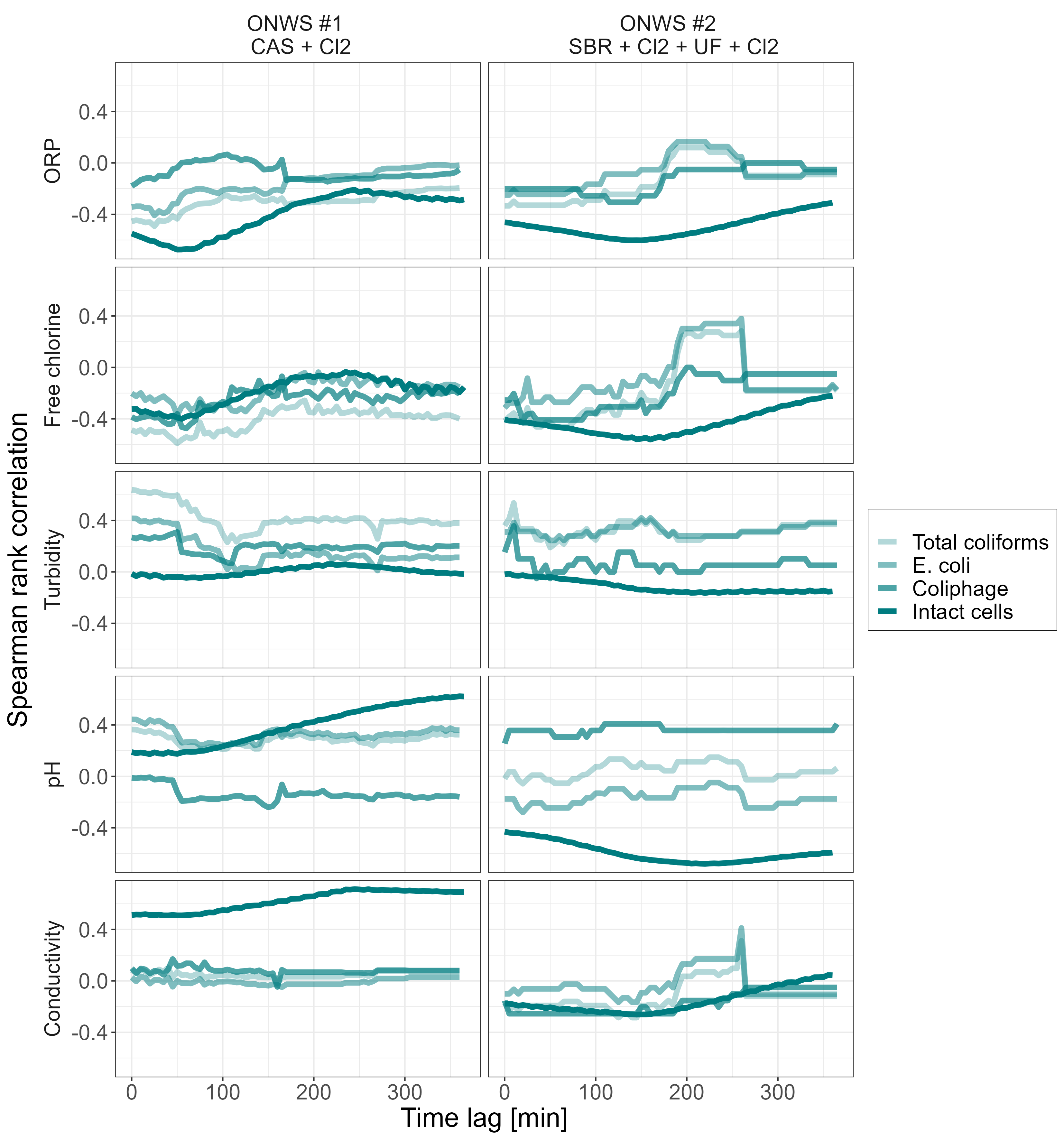


**Figure 1.** Spearman rank correlation between sensor measurements and microbial water quality for time lags between 0 and 300 min. ONWS: on-site non-potable water system. CAS: conventional activated sludge. Cl_2_: chlorine. SBR: sequencing batch reactor. UF: ultrafiltration. ORP: oxidation-reduction potential.

**Supplementary Information 3: Logistic Regression Analysis**

Following one of the methods suggested in Reynaert et al. (2023), Figure 2 exemplifies an approach to define sensor setpoints that ensure meeting a log_10_-ICC < 5 in OWNS#2-TF based on logistic regressions with and without time lag. We see that the mean ORP required to be 95% confident to meet the log_10_-ICC target is higher than the maximum ORP measurements without accounting for a potential time lag between ORP and ICC measurements (Figure 2.A). This requirement for a very high ORP setpoint to ensure meeting the defined ICC threshold is likely due to the time lag between sensor measurements and microbial water quality: for instance, it may take some time for the ICC to increase after the ORP has decreased. The effect of these dynamics can be partially reduced by computing the logistic regression for the time-lagged data, as presented in Figure 2.B (time lag of 150 min, corresponding to the time lag for which the correlation between ICC and ORP is highest). Incorporating the time lag results in a higher confidence of prediction and smaller confidence band. With this approach, ORP setpoints are 740 mV (mean), respectively 790 mV if we consider the 95% confidence interval of the prediction. These values are a bit higher but comparable to those computed in Reynaert et al. (2023) (690 mV mean, 720 mV confidence interval), but in a similar range. To validate the sensor setpoints from Reynaert et al. (2023) it will, however, be necessary to collect a suitable dataset in systems that achieve a stable water quality.

**
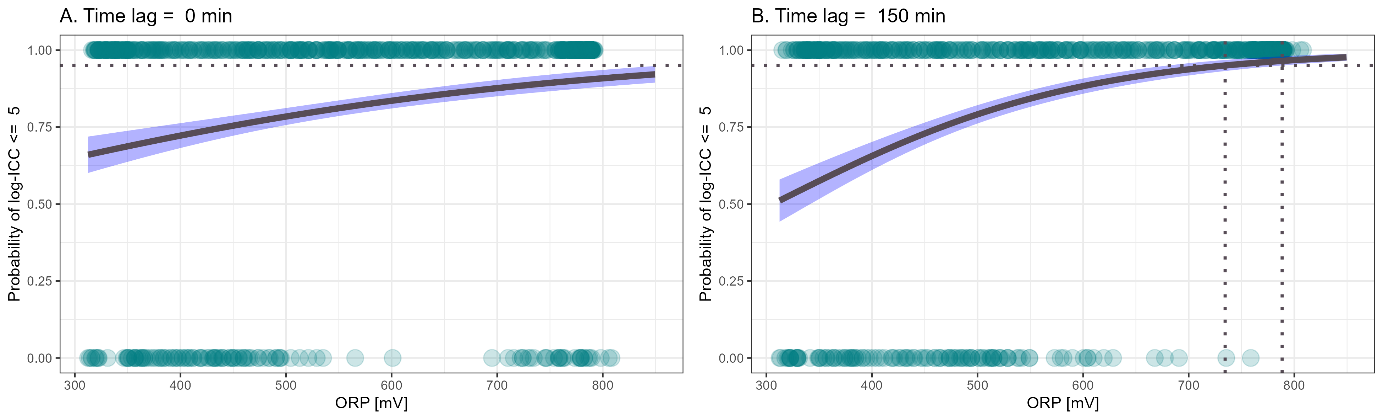
**

**Figure 2.** Logistic regressions show the probability of meeting a log_10_-concentration of intact cells of 5 as a function of the oxidation-reduction potential (ORP), without (A) and with (B) time lag for on-site water system #2 (water reused for toilet flushing) The time lag of 150 min is the time lag corresponding to the maximum correlation (see Figure 1). Blue shaded interval: 95% confidence interval of logistic regression. Horizontal dashed line: 95% probability. Vertical dashed lines: ORP required to be 95% that the water quality target is met (in average, or considering the 95% confidence interval).

**References**

Reynaert, E., Greenwood, E., Ndwandwe, B., Riechmann, M., Udert, K., & Morgenroth, E. (2020). Practical Implementation of True on-Site Water Recycling Systems for Hand Washing and Toilet Flushing. *Water Research X, 100051*.

Reynaert, E., Gretener, F., Julian, T. R., & Morgenroth, E. (2023). Sensor Setpoints That Ensure Compliance with Microbial Water Quality Targets for Membrane Bioreactor and Chlorination Treatment in on-Site Water Reuse Systems. *Water Research X, 18*, 100164.
